# Supplementary material for: Persistent spatial clustering and predictors of pediatric La Crosse virus neuroinvasive disease risk in eastern Tennessee and western North Carolina, 2003–2020
Source: PLoS Negl Trop Dis. 2024 Jun 6;18(6):e0012186. doi: 10.1371/journal.pntd.0012186 (PMC11156276; doi:10.1371/journal.pntd.0012186)
Supplement: S1 Table — (DOCX) [file pntd.0012186.s001.docx]

| **S1 Table.** Data sources, temporal resolution, spatial resolution, and usage. | | | | |
| --- | --- | --- | --- | --- |
| Source | Data | Year(s) | Resolution | Usage |
| Tennessee Department of Health | Cases of probable and confirmed La Crosse virus neuroinvasive disease in Tennessee | 2003 to 2020 | Home address | Cluster investigation, predictor investigation |
| North Carolina Department of Health and Human Services | Cases of probable and confirmed La Crosse virus neuroinvasive disease in North Carolina | 2003 to 2020 | Home address | Cluster investigation, predictor investigation |
| United States Census Bureau Decennial Survey | Total population under the age of 18 | 2000, 2010, 2020 | ZCTA (standardized to 2010 ZCTAs) | Cluster investigation |
| United States Census Bureau TIGER/Line Files | Cartographic boundaries | 2010, 2020 | State, County, ZCTA | Maps, cluster investigation, predictor investigation |
| United States Census Bureau 5-year American Community Survey | Total population under 20 years, percentage of population under 20 years that is male, vacant housing density, population density, percentage of population in poverty | 2020 | 2020 ZCTAs | Predictor investigation |
| National Land Cover Database (NLCD) | Percentage of developed land, percentage of forested land, change in percentage of developed land from 2001 to 2019 | 2001, 2019 | 30m (transformed to percent land cover for calculated for 2020 ZCTAs) | Predictor Investigation |
| PRISM Climate Group | Average temperature (°C), cumulative precipitation (mm), (°C), | August averages from 2015–2020 | 4km (transformed to mean values for 2020 ZCTAs) | Predictor Investigation |
| United States Geographic Service LiDAR Explorer | Digital Elevation Map (DEM) | Varies | 30m (transformed to median elevation for 2020 ZCTAs) | Predictor Investigation |
