# Supplementary material for: Persistent spatial clustering and predictors of pediatric La Crosse virus neuroinvasive disease risk in eastern Tennessee and western North Carolina, 2003–2020
Source: PLoS Negl Trop Dis. 2024 Jun 6;18(6):e0012186. doi: 10.1371/journal.pntd.0012186 (PMC11156276; doi:10.1371/journal.pntd.0012186)
Supplement: S2 Table — (DOCX) [file pntd.0012186.s002.docx]

| **S2 Table.** Potential predictor variables, rationale for inclusion, and associated references. | | |
| --- | --- | --- |
| **Potential Predictor Variable** | **Rationale for inclusion** | **Associated references** |
| Percentage population below poverty line, education, and age of housing (i.e., socioeconomic status) | Previously identified associations with population-level risk | [1,2] |
| Percentage of vacant housing | Vacant housing tends to be associated with increased availability of mosquito habitat and mosquito abundance | [3,4] |
| Population density | Hypothesis that low population density is associated with less anthropogenic development, supporting abundance of native eastern tree hole mosquito (principal LACV vector) based on prior association of *Aedes triseriatus* with hardwood forests. Previously associated with population-level risk in West Virginia from 2003-2007. | [2] |
| Percentage of under-20 population that is male | Included as a potential confounder because more than half of reported LACV-ND cases are males. | [5] |
| Percentage of developed land and percentage of forest land (i.e., land cover) | Expected to be associated with relative abundances of native and accessory vectors, with anthropogenic development favoring invasive vectors, *Aedes albopictus* and *Ae. japonicus japonicus* and with forested areas favoring the native and principal vector, *Aedes triseriatus*. | [6–10] |
| Change in percentage of developed land | Included based on prior hypotheses that human encroachment into previously undeveloped areas (i.e., land use change) could support emergence of LACV-ND in focal populations | [10] |
| Temperature, precipitation, and dew point | Based on substantial evidence for the role of broad and local climate patterns on vector development and pathogen transmission. The interactions of temperature, precipitation, and ambient moisture influence vector development rates and pathogen transmission rates. | [11–14] |
| Elevation | Assessed because of its potential to account for climate effects beyond those included in the study, and due to substantial elevation differences throughout the study. | [11–14] |

**References**

1. Day CA, Odoi A, Fryxell RT. Geographically persistent clusters of La Crosse virus disease in the Appalachian region of the United States from 2003 to 2021. PLoS Negl Trop Dis. 2023;17: e0011065. doi:10.1371/journal.pntd.0011065

2. Haddow AD, Bixler D, Schuh AJ. The demographic and socioeconomic factors predictive for populations at high-risk for La Crosse virus infection in West Virginia. Braga, editor. PLoS ONE. 2011;6: e25739. doi:10.1371/journal.pone.0025739

3. Becker B, Leisnham P, LaDeau S. A tale of two city blocks: differences in immature and adult mosquito abundances between socioeconomically different urban blocks in Baltimore (Maryland, USA). Int J Environ Res Public Health. 2014;11: 3256–3270. doi:10.3390/ijerph110303256

4. Barrera R, Acevedo V, Amador M. Role of abandoned and vacant houses on *Aedes aegypti* productivity. Am J Trop Med Hyg. 2021;104: 145–150. doi:10.4269/ajtmh.20-0829

5. Vahey GM, Lindsey NP, Staples JE, Hills SL. La Crosse virus disease in the United States, 2003–2019. Am J Trop Med Hyg. 2021;105: 807–812. doi:10.4269/ajtmh.21-0294

6. Nasci RS, Moore CG, Biggerstaff BJ, Panella NA, Liu HQ, Karabatsos N, et al. La Crosse encephalitis virus habitat associations in Nicholas County, West Virginia. J Med Entomol. 2000;37: 559–570. doi:10.1603/0022-2585-37.4.559

7. Bevins SN. Establishment and abundance of a recently introduced mosquito species *Ochlerotatus japonicus* (Diptera: Culicidae) in the southern Appalachians, USA. J Med Entomol. 2007;44: 945–952. doi:10.1603/0022-2585(2007)44

8. Haddow AD, Gerhardt RR, Jones CJ, Odoi A. The mosquitoes of eastern Tennessee: studies on abundance, habitat preferences, and host-seeking behaviors. J Vector Ecol. 2009;34: 70–80. doi:10.1111/j.1948-7134.2009.00009.x

9. Tamini TT, Byrd BD, Goggins JA, Sither CB, White L, Wasserberg G. Peridomestic conditions affect La Crosse virus entomological risk by modifying the habitat use patterns of its mosquito vectors. J Vector Ecol. 2021;46: 34–47.

10. Leisnham PT, Juliano SA. Impacts of climate, land use, and biological invasion on the ecology of immature *Aedes* mosquitoes: implications for La Crosse emergence. EcoHealth. 2012;9: 217–228. doi:10.1007/s10393-012-0773-7

11. Campbell-Lendrum D, Manga L, Bagayoko M, Sommerfeld J. Climate change and vector-borne diseases: what are the implications for public health research and policy? Philos Trans R Soc B Biol Sci. 2015;370: 20130552. doi:10.1098/rstb.2013.0552

12. Evans MV, Hintz CW, Jones L, Shiau J, Solano N, Drake JM, et al. Microclimate and larval habitat density predict adult *Aedes albopictus* abundance in urban areas. Am J Trop Med Hyg. 2019;101: 362–370. doi:10.4269/ajtmh.19-0220

13. Murdock CC, Evans MV, McClanahan T, Miazgowicz K, Tesla B. Fine-scale variation in microclimate across an urban landscape changes the capacity of *Aedes albopictus* to vector arbovirus. PLoS Negl Trop Dis. 2016;11: e0005640. doi:10.1101/090613

14. Alto BW, Juliano SA. Precipitation and temperature effects on populations of *Aedes albopictus* (Diptera: Culicidae): implications for range expansion. J Med Entomol. 2001;38: 646–656. doi:10.1603/0022-2585-38.5.646
