# Supplementary material for: Persistent spatial clustering and predictors of pediatric La Crosse virus neuroinvasive disease risk in eastern Tennessee and western North Carolina, 2003–2020
Source: PLoS Negl Trop Dis. 2024 Jun 6;18(6):e0012186. doi: 10.1371/journal.pntd.0012186 (PMC11156276; doi:10.1371/journal.pntd.0012186)
Supplement: S3 Table — (DOCX) [file pntd.0012186.s003.docx]

| **S3 Table.** Descriptive statistics of potential predictor variables. | | | |
| --- | --- | --- | --- |
| **Predictor** | **Mean**  **(SD^1^)** | **Median**  **(IQR^2^)** | **Min, Max** |
| Mean average temperature in August (°C) | 23.4  (1.7) | 23.8  (2.3) | 19.1, 26.5 |
| Mean cumulative precipitation in August (mm) | 135.6  (28.0) | 128.9  (28.8) | 88.3, 241.2 |
| Percentage of forested land in 2019 (per 10%) | 57.6  (24.5) | 60.6  (38.8) | 0, 95.9 |
| Change in % developed land from 2001 to 2019 | 1.25  (1.7) | 0.59  (1.14) | 0, 10.7 |
| Population density per km^2^ | 93.0  (168.1) | 29.9  (71.9) | 0.7, 1429.8 |
| Percentage of vacant housing | 22.2  (17.1) | 16.4  (16.6) | 0, 97.3 |
| Percentage of houses built before 1969 | 29.8  (15.4) | 26.9  (17.2) | 0, 100 |
| Percentage of population with less than a high school education | 14.6  (10.8) | 13.6  (9.8) | 0, 100 |
| Percentage of households in poverty | 15.7  (9.7) | 14.5  (10.2) | 0, 100 |
| Percentage of under-20 population that are males | 51.6  (12.4) | 51.6  (9.5) | 0, 100 |
| ^1^Standard Deviation  ^2^Interquartile Range | | | |
