# Supplementary material for: Persistent spatial clustering and predictors of pediatric La Crosse virus neuroinvasive disease risk in eastern Tennessee and western North Carolina, 2003–2020
Source: PLoS Negl Trop Dis. 2024 Jun 6;18(6):e0012186. doi: 10.1371/journal.pntd.0012186 (PMC11156276; doi:10.1371/journal.pntd.0012186)
Supplement: S4 Table — (DOCX) [file pntd.0012186.s004.docx]

| **S4 Table.** Results of univariable global negative binomial regression models (p-values < 0.20 in bold). | | | |
| --- | --- | --- | --- |
| Predictor | Coefficient | 95% Conf. Int.^1^ | p-value |
| Mean avg temp in August (°C) 2015–2020 | -0.44 | -0.58, -0.30 | **< 0.001** |
| Mean precipitation in August (mm) 2015–2020 | 0.01 | 0.001, 0.02 | **0.04** |
| Percentage of forested land in 2019 (per 10%) | 0.02 | -0.3, -0.06 | **< 0.001** |
| Change in percentage of developed land from 2001 to 2019 | -0.2 | -0.4, -0.1 | **0.01** |
| Percentage of vacant housing | 0.05 | 0.03, 0.06 | **< 0.001** |
| Percentage of houses built before 1969 | -0.02 | -0.04, 0.005 | **0.1** |
| Percentage of population with less than a high school education | 0.007 | -0.04, 0.05 | 0.73 |
| Percentage of households in poverty | -0.03 | -0.08, 0.009 | **0.1** |
| Percentage of under-20 population that are male | 0.05 | -0.03, 0.04 | 0.8 |
| ^1^Confidence Interval | | | |
