# Supplementary material for: Persistent spatial clustering and predictors of pediatric La Crosse virus neuroinvasive disease risk in eastern Tennessee and western North Carolina, 2003–2020
Source: PLoS Negl Trop Dis. 2024 Jun 6;18(6):e0012186. doi: 10.1371/journal.pntd.0012186 (PMC11156276; doi:10.1371/journal.pntd.0012186)
Supplement: Attachment — Submitted filename: Response to reviewers.docx [file pntd.0012186.s005.docx]

**Description of major revisions based on reviewer comments:**

Introduction:

- Significant expansion of the introduction section, including mention of accessory vectors and economic toll of LACV-ND in the first paragraph, an expanded second paragraph on the geographic expansion of LACV-ND into Appalachia, and new paragraphs that highlight the need for spatial cluster and risk analysis to guide prevention in the Appalachian region.

Methods:

- Revised methods section to reduce confusion regarding the use of case data from 2015-2020 and associated climate data in the predictor investigation
- Added a new supplementary table containing rationale for each predictor variable and associated in-text citations. Text that is redundant with this table was removed from the methods for conciseness.

Results:

- Revised Table 1 with age categories that more closely reflect the analyses in the manuscript
- Investigated dew point as a potential predictor in response to requests for the inclusion of humidity
  - Did not include dew point in final models because of its close correlation with temperature.
- Moved Table 3 to Supplementary Information

Discussion:

- Added a paragraph to the discussion section detailing limitations of geographically weighted regression

Direct responses to reviewer comments are included in the pages below. Reviewer comments are in **red** and author responses are in **blue.**

**Reviewer #1:**

***Methods***

- *Are the objectives of the study clearly articulated with a clear testable hypothesis stated?*
- *Is the study design appropriate to address the stated objectives?*
- *Is the population clearly described and appropriate for the hypothesis being tested?*
- *Is the sample size sufficient to ensure adequate power to address the hypothesis being tested?*
- *Were correct statistical analysis used to support conclusions? Are there concerns about ethical or regulatory requirements being met?*

1. **This study used data from 2023-2022. Why climate data used only 2015-2020?**

This study used data from 2003-2020 for the cluster investigation, and the predictor investigation only included cases from 2015-2020 to identify predictors that were associated with the most recent distribution of cases. This period also reflects the most recent case definition for La Crosse virus neuroinvasive disease. The temporal range of climate data was selected to match the case data.

**2. Why focus on 18 years and under? Why page 6 line 142 change to 19 years and younger?**

We focused on the pediatric population based on existing knowledge that LACV-ND is primarily a disease of children. Disease in adults is also likely confounded by higher rates of other bunyavirus infections (e.g., Jamestown Canyon virus) that are not easily differentiated. So in addition to being the predominant risk group, using this criteria increases specificity.

Regarding the use of age 19 years and younger for the predictor investigation, this decision was related to data availability. The 2020 5-year American Community Survey, from which the 2020 underlying population data was taken, only includes five-year age groups, so the 19 and under age group.

We have elaborated on this decision in the revised methods section. Please note that there was only one case aged 19, so this decision did not have a substantial effect on the sample size of the study.

***Results***

- *Does the analysis presented match the analysis plan?*
- *Are the results clearly and completely presented?*
- *Are the figures (Tables, Images) of sufficient quality for clarity?*

**1. In the study area was 486 cases but male 275 + female 198 = 473. What happen to other 13 cases? Page 11 line 283-285**

Thank you for noticing this. The missing 13 cases were ones where the sex was not reported. We clarified this in the revised manuscript.

**2. In the method section focus on 18 years and younger, why in the result become 15 years and younger? What is the criteria to classified age groups in table 1?**

With consideration of your comment, we revised Table 1 to better align with the other analyses in the manuscript. It now summarizes ages 0-9 and 10-19.

***Conclusions***

- *Are the conclusions supported by the data presented?*
- *Are the limitations of analysis clearly described?*
- *Do the authors discuss how these data can be helpful to advance our understanding of the topic under study?*
- *Is public health relevance addressed?*

**No Response**

*Editorial and Data Presentation Modifications?*

**No Response**

*Summary and General Comments*

**The authors present an analysis during 2003 to 2022 of data on La Crosse virus. Overall the paper is well written; however, there are a few points that need to be reassessed and strengthened.**

Thank you for your helpful comments. We addressed your concerns in the revised manuscript.

**Reviewer #2:**

***Methods***

- *Are the objectives of the study clearly articulated with a clear testable hypothesis stated?*
- *Is the study design appropriate to address the stated objectives?*
- *Is the population clearly described and appropriate for the hypothesis being tested?*
- *Is the sample size sufficient to ensure adequate power to address the hypothesis being tested?*
- *Were correct statistical analysis used to support conclusions? Are there concerns about ethical or regulatory requirements being met?*

**Yes. The study design is appropriate. I recommend outlining in detail some limitations of Geographically Weighted Regression in the limitation section of the manuscript.**

Thank you. We have added language to the discussion section to address limitations of geographically weighted regression.

***Results***

- *Does the analysis presented match the analysis plan?*
- *Are the results clearly and completely presented?*
- *Are the figures (Tables, Images) of sufficient quality for clarity?*

**Yes.**

***Conclusions***

- *Are the conclusions supported by the data presented?*
- *Are the limitations of analysis clearly described?*
- *Do the authors discuss how these data can be helpful to advance our understanding of the topic under study?*
- *Is public health relevance addressed?*

**Yes. I recommend outlining limitations of Geographically Weighted Regression in the limitation section**
Thank you. We outlined limitations of GWR in the discussion.

***Conclusions***

- *Are the conclusions supported by the data presented?*
- *Are the limitations of analysis clearly described?*
- *Do the authors discuss how these data can be helpful to advance our understanding of the topic under study?*
- *Is public health relevance addressed?*

**The study was well organized and well executed, and the results make a nice contribution to ongoing efforts to understand patterns of LaCrosse encephalitis virus risk and correlations with socioeconomic and environmental data. The models and results are appropriate for the analyses, with one caveat regarding geographically weighted regression, which could be outlined in the limitations section of the manuscript.

Specifically, there has been some debate in the literature about whether GWR is more of a descriptive modeling approach and what this means when inferring model results. I recommend outlining in the study limitations some of the limitations of GWR and pointing out that additional modeling frameworks are also available to analyze spatial areal data, including conditional autoregressive (CAR) models and simultaneous autoregressive models (SAR) models.**

We agree that the best use of GWR is as descriptive modeling approach, and we added text that details this limitation. In this study, we used GWR to provide a better understanding of spatial variation in the relationship between predictors (identified with a global model) and the outcome, which we think fits within the applicability of the approach.

**Other than this caveat, I have only minor suggestions for clarity to help improve readability of the manuscript:**

**Under the software heading, spatial scan statistics are not mentioned and only descriptive statistics are associated with R.**

Thank you for noticing this error. We revised this section to clarify that R was used to implement the flexible scan statistic and added SaTScan as an additional software.

**Under the heading Global Model Fitting, it would be helpful to the reader to clarify here and indicate that this is the first part of the geographically weighted regression (GWR). Please state that a generalized linear model or a generalized linear mixed effects model (negative binomial) was used.**

We agree that our approach to model fitting was unclear in the original manuscript. We revised this section accordingly. It now clarifies the three-step model building process for the global model, which is used to define the local model.

***Summary and General Comments***

**The objective of this study is to analyze the spatiotemporal distribution of pediatric LaCrosse Encephalitis virus neuroinvasive disease between 2003 and 2020 in the Appalachian Mountains region of Tennessee and western North Carolina where consistent transmission occurs. The authors used multiple methods to investigate spatial clusters and space-time clusters, and they used a geographically weighted regression to investigate effects of covariates on observed patterns. All analyses were performed at the zip code tabulation area level.

The study was well organized and well executed, and the results make a nice contribution to ongoing efforts to understand patterns of LaCrosse encephalitis virus risk and correlations with socioeconomic and environmental data. The models and results are appropriate for the analyses, with one caveat regarding geographically weighted regression, which could be outlined in the limitations section of the manuscript.**

Thank you. We addressed your concerns in the discussion section.

**Reviewer #3:**

***Methods***

- *Are the objectives of the study clearly articulated with a clear testable hypothesis stated?*
- *Is the study design appropriate to address the stated objectives?*
- *Is the population clearly described and appropriate for the hypothesis being tested?*
- *Is the sample size sufficient to ensure adequate power to address the hypothesis being tested?*
- *Were correct statistical analysis used to support conclusions? Are there concerns about ethical or regulatory requirements being met?*

**1-Land cover data methods section- It is unclear why land change from 2001 to 2019 was used, when the case data was from 2003-2020. Please correct accordingly or clarify rationale for temporal variation.**

The National Land Cover database only has land cover datasets for select years, with the earliest in 2001 and 2019 being the most recent available year within the study period. We used land cover change from 2001-2019 because 2001 was the earliest available land cover dataset, so the 2001-2019 range captured the most possible land cover change.

**2-Climate data methods section- It is unclear why climate data for five years was used when case data was for 17 years. Again, please correct accordingly or clarify rationale for temporal variation. Note: PRISM data goes back decades allowing for overlapping datasets (climate and case).**

The temporal range of the climate data was selected to match the case data in the predictor investigation, which was from 2015-2020. In the revised manuscript, we reorganized the methods section to increase clarity for readers regarding this point. In the original version, the temporal range of the predictors is mentioned early in the methods to define the sources of the datasets.

In consideration of your comments, we moved that text to the predictor investigation section so that readers can first see that we used cases from 2015-2020 for the predictor investigation before seeing the temporal range of the predictors.

**3-Climate data methods section- Why is humidity not included in this data set? Humidity is arguably just as important in mosquito biology as temperature and precipitation. Please re-rerun the analyses with this factor, or provide rationale as to why it was not included.** [**https://www.ncbi.nlm.nih.gov/pmc/articles/PMC10299817/**](https://www.ncbi.nlm.nih.gov/pmc/articles/PMC10299817/)

PRISM does not provide historical humidity data, but it does provide dew point, which is closely related. In consideration of your comment, we calculated mean dew point in August from 2015-2020 using methods identical to the ones we originally used for temperature and precipitation. The mean dew point was strongly correlated with temperature (r = 0.94), so we did not include it in the models. As humidity is largely a function of dew point and temperature, it is likely that humidity is closely correlated with temperature in this study area as well, which would prevent it from providing new information beyond what is already captured in the original models. We included this update in the revised methods and results.
**4-Land cover, climate and elevation predictors methods section- The rationale for using a one year snapshot of land cover classification types is unclear. Why would forest land cover in 2019 have any prediction relationship with cases in 2003? If you are using cumulative incidence as a measure, this should be clarified.**

The case data used in the predictor investigation was only from 2015-2020. Within the period of 2015-2020, land cover data from NLCD is only available from 2016 and 2019. We chose to use the most recent that was within the range of cases. We revised the text to clarify the differences in temporal range between the cluster and predictor investigations earlier in the manuscript.

Regarding your mention of cumulative incidence: please note that we are essentially using cumulative incidence as the outcome variable. We used the total number of cases from 2015-2020 as the outcome variable, but specified the total population as the offset, effectively modeling cumulative incidence. This was described in the original article, but we added some clarifying text in the revised version under “Methods: Predictor Investigation-Global Model Selection” to improve clarity.

***Results***

- *Does the analysis presented match the analysis plan?*
- *Are the results clearly and completely presented?*

*Are the figures (Tables, Images) of sufficient quality for clarity?*
 **(No Response)**

***Conclusions***

- *Are the conclusions supported by the data presented?*
- *Are the limitations of analysis clearly described?*
- *Do the authors discuss how these data can be helpful to advance our understanding of the topic under study?*
- *Is public health relevance addressed?*

**No response.**

***Editorial and Data Presentation Modifications?***

**Minor edits:

1-The introduction could be strengthened with more descriptive facts. The introduction comes across very vague and fails to 'sell' the reader on the importance and value of this article. For example, you could state "LACV has grave economic consequences--a single case can accrue up to $3 million in projected costs--a particularly concerning impact considering socioeconomically disadvantaged populations are at greatest risk for this disease."  Ref:** [**https://pubmed.ncbi.nlm.nih.gov/14695088/**](https://pubmed.ncbi.nlm.nih.gov/14695088/) **and** [**https://pubmed.ncbi.nlm.nih.gov/21980533/**](https://pubmed.ncbi.nlm.nih.gov/21980533/)

We added a sentence to the opening paragraph addressing the potential economic toll of LACV-ND. The original introduction addresses the fact that most cases occur in the socioeconomically disadvantaged Appalachian region.

**2- IRB approval for TN is noted, but not NC. If NC health department did not require internal IRB approval, this should be stated to clarify ethical approval processes.**

North Carolina relied on the university (UTK) IRB approval as part of the overriding data use agreement. For Tennessee, the university initiated a reliance agreement with the TN Department of Health based on their IRB approval. For North Carolina, we received IRB approval from UTK, and the NC DHHS used that to initiate a data use agreement. As suggested, we revised this section for clarity.

**3-Lines 153-155. Please describe the extent of 'developed land' used. National remote sensing standards use five 'developed' land classifications (see link). Did you consider any change in development category apart of 'percent change in developed land' or what metric did you use?** [**https://www.mrlc.gov/data/legends/national-land-cover-database-class-legend-and-description**](https://www.mrlc.gov/data/legends/national-land-cover-database-class-legend-and-description)**.**

The developed land categories are described in the original manuscript in lines 152-153. Specifically, “low intensity, medium intensity, and high intensity” were aggregated to create the single category used here. We omitted the category “developed, open space”, and the NLCD does not have a fifth category for developed land.

We measured change in developed land from 2001 to 2019 and used a static variable of percentage developed land in 2019, but no other changes in land cover were investigated. Please note that percentage of developed land was highly correlated with both population density (r=0.89) and percentage of forested land (r=-0.77) (lines 371-372 in the original manuscript under the results subsection **Predictor Investigation – Global model**). Consequently, we chose to omit the percentage developed land predictor in favor of population density and percentage forested land (rather than drop those two predictors instead).

**4-Lines 239-240: Authors mention an important point and study limitation. Perhaps using underlying Ecoregion IV would be a better indicator of environment than 'forest'. Forest is defined as three different categories per national remote sensing standards (which forest type should be clarified), yet none of these get to the detail level of at-risk individual tree species ecology.** [**https://www.mrlc.gov/data/legends/national-land-cover-database-class-legend-and-description**](https://www.mrlc.gov/data/legends/national-land-cover-database-class-legend-and-description)

The forest types used in this study are clarified in the original manuscript in lines 151-152 – each of the three categories are aggregated into a single category referred to here as “forest”. The primary goal was to determine whether there are associations between land cover and risk at this scale, with the understanding that in this study area, developed areas are comprised of less forested land and vice versa (see previous comment – developed land and forested land are strongly correlated in this study area). Although we agree that additional research at a finer scale could benefit from a more detailed assessment of forest types, we feel that the land cover categories used here are adequate for this study given the ZIP code level human data. We revised the text to more explicitly describe the categories included in the Forest and Developed Land categories, and added a reference corresponding to the official NLCD land cover categories.

**5-Results section: Overall this section reads very verbose. I would encourage the senior author(s) to refine and streamline this section.

6-Table 3 (line 351): Given the large number of tables in the paper, this one would make a good candidate for a supplemental table OR authors could combine tables 3 and 4.**

We consider Table 3 important because we refer to the weak associations between socioeconomic variables and the outcome in the discussion. However, because the results section is lengthy and Table 3 is not necessary for interpreting the primary results, we you’re your advice and moved Table 3 to Supporting Information.

**7-Lines 362-363: I am not surprised you only found a small number of zipcodes with statistically significant associations. This would likely change if you used the same years for both datasets--see prior comments on non-matching temporal datasets.**

The case data and climate data had the same temporal range – see prior responses. The lack of areas with strong associations is more likely due to the limited range of cases, which primarily occur in a few ZCTAs. The global model indicates that climate variables are broadly associated with risk, but the GWR indicates that strength of those associations was not uniform throughout the study area.

**8-Limitation: Authors fail to mention that Aedes japonicus is another important LACV vector with a differing ecology than Aedes triseratus. The ecology for this secondary vector is not evaluated by the current paper's methods and future studies should evaluate this additional vector.**

There are several accessory LACV vectors, *Ae. j. japonicus* being among them. The predictors investigated here would certainly be relevant to the ecology of other vectors – i.e., climatic factors, land cover, and socioeconomic metrics. Additional site-level research to untangle their relationship with LACV transmission is warranted to uncover important relationships with *Ae. j. japonicus* and other accessory vectors, but we respectfully disagree that the ecology *Ae. j. japonicus* represents a limitation of this study.

We added text referring to *Ae. albopictus* and *Ae. j. japonicus* (considered the most-likely accessory vectors) to the introduction and broadened some writing in the methods to imply that land cover predictors correspond to each of the LACV vectors.

**9-Figure 5: The legend is incorrect. Your low-risk clusters all list '2003-2018'.**

The legend is correct. SaTScan identifies space-time clusters that comprise at most 90% of the study period (see lines 207-208 in the original manuscript). In this case, clusters were identified for the maximum 90% of the study period for each of the low risk clusters – i.e., 2003-2018.


***Summary and General Comments***
**The authors perform spatial modelling to identify persistent areas of pediatric La Crosse encephalitis. While rare, this disease has important mortality and morbidity implications and leveraging geospatial techniques has promise to inform public health interventions. The authors are particularly applauded for including key state public health officials, who have the capacity to employ these models into their respective state's public health activities.

Some core methodological issues inhibit the rigor and potential validity of the current analysis. These issues are all easily addressable, and pending their incorporation would make for an appropriate article that would be a welcomed addition to the scientific literature. Detailed major and minor comments are listed in prior reviewer sections.**
