# Supplementary material for: Persistent spatial clustering and predictors of pediatric La Crosse virus neuroinvasive disease risk in eastern Tennessee and western North Carolina, 2003–2020
Source: PLoS Negl Trop Dis. 2024 Jun 6;18(6):e0012186. doi: 10.1371/journal.pntd.0012186 (PMC11156276; doi:10.1371/journal.pntd.0012186)
Supplement: Attachment — Submitted filename: reviewer comments.docx [file pntd.0012186.s006.docx]

Reviewers 2 and 3 did not suggest additional revisions, so only comments from reviewer 4 are addressed here. Below is a summary of revisions to the manuscript:

- Based on reviewer comments, we added a table showing the results of a maximal multivariable regression model for the predictor investigation. This provides additional transparency to the analysis by showing that only the climatic variables were significant in the multivariable model. We assessed a multimodel selection approach as suggested by the reviewer (see comment below), but it did not change the results, so we opted not to describe it in the manuscript.
- The methods are more clearly divided by two objectives. Proper citations for our methods (and related parameters), previously cited at the first description of each approach, were inserted throughout paragraphs.
- The discussion section contains an added paragraph addressing a hypothesized mechanism for the focal persistence of LACV. We also made minor revisions to the introduction and discussion to address the exploratory nature of the predictor investigation and the limitation associated with our choice of climate variables.

**Reviewer's Responses to Questions**

Reviewer #4: This study presents a spatial analysis of pediatric La Crosse virus cases in an area with persistently high disease activity. Overall, the study is well implemented and will provide important guidance for public health planning and vector control activities in the study region. I also thought the authors provided a very nice description of the rationale behind user-specified parameters in the SaTScan analysis, which could serve as a model for other studies adopting this methodology.

That said, I do have some major (and minor) concerns that the authors should address to strengthen the manuscript.

**Major Comments**

The Methods section would greatly benefit from considerable revision, mostly for cohesion, flow, and organization. Need to give definitions/explanations/rationale behind choices and data products before they are mentioned in text; as written, many sections felt "out of order" from the reader perspective. Concepts and terms are used in the manuscript, but introduced or expanded upon in later sections. For example, the description of switching rates to 19 years and under occurs much later in the methods (in the predictor investigation section), but is seemingly referenced in one of the cluster analyses. In general, I think many smaller sections in the methods could be combined, cohesively outlining the preparation of data inputs, then describing how each dataset was used for each statistical analysis. As written, the methods jump around, and it becomes difficult for the reader to follow which data are feeding into which analysis. Method choices are also under cited in many
instances (i.e., provide citations for things like parameter choices, threshold choices, etc).

We have slightly reorganized the methods section. Mainly, we more clearly split it by objective (objective 1 and objective 2). We chose to summarize the data preparation for each objective separately rather than at the beginning of the methods, because summarizing all of the data preparation at the beginning (which we did in the first submission) seemed to create a different confusion, largely because of the differences in time periods being investigated. Additional citations were also added throughout the methods.

Curious as to why ‘rflexscan’ was used to implement purely spatial clusters, when SaTScan (which is used to implement the space-time scan statistic) also has this option. Was there a particular reason why Tango’s restricted flexibly shaped scan statistic was used? This is not a commonly used test, and I think it would be beneficial to briefly describe what this is doing, and why you chose to use it here.

Tango’s restricted flexibly shaped scan statistic was used because it is more sensitive to clusters of non-circular shapes than the circular scanning statistic that is implemented in FleXScan. Although SaTScan (i.e. the circular scan statistic) is more commonly used, there often is no reason to expect clusters to be circular. In this study, we presumed that clusters were more likely to assume arbitrary shapes. Unfortunately, we are not aware of software that conveniently implements a flexibly shaped space-time scan statistic, so we used SaTScan for that analysis. In this case, both options (flexible vs. circular or spatial vs. space-time) yielded very similar outputs, which we think strengthens our conclusions.

My biggest issues were with choices made in the implementation of the Predictor Investigation (specifically the two points below). This section should be revised, or could even be omitted given the strength of the other findings.

L275 Climate variables were chosen for the month of August because of peak cases, but what about potential lags? I.e., might climate in the months preceding peaks of cases influence transmission via upstream influence on vector reproduction, or host populations? What is the mechanism of transmission that is being proposed by limiting to climate in August? (i.e., are you saying that climate in the month of transmission is saying something about the vector biology, transmission, human behavior that makes people susceptible to infectious bites?).

We agree climate lags very likely influence transmission through the mechanisms that you described. However, we do not think the modeling approach used here, with five years of data aggregated as ZCTAs, is sufficient to understand the relative effects of climate at different points in the year on transmission. Climate in August provided a basic summary of the climate tendencies of the study area, and provided relationships which were strong enough to mediate the effects of socioeconomic factors that were identified as predictors of high-risk areas in previous studies.

In consideration of your comments, we reanalyzed the global model using the mean temperature and precipitation from May–August from 2015–2020 to see if capturing climate values from a larger proportion of the transmission period would substantially alter our results (these results are not presented in the revised manuscript). The outcome was very similar to our original analysis; precipitation and temperature had positive but antagonistic associations with cumulative incidence, and climatic variables were the only significant predictors in multivariable models. These results are not presented in the revised manuscript.

We do agree that this approach is limited in general, and additional research is needed that investigates transmission mechanisms at a finer scale using vector and host infection data, as we described in the discussion section. We revised Objective 2 and some writing in the discussion section to emphasize that the predictor investigation in this study is largely exploratory. Despite the limitations, this is currently the best effort to associate the distribution of LACV-ND cases with climate. The primary value of this finding may not be the association between August climate and risk specifically, but the evidence that climate may mediate the effects of sociodemographic factors associated with LACV-ND in previous studies. We hope that this study will be a useful starting point for grant proposals that can better investigate this question.

L304 stepwise model selection procedures are notoriously problematic, often leading to locally (but not globally) optimal solutions. Suggest trying a multimodel selection framework, with an appropriate information criterion for ranking models, and seeing if this changes your final model.

We recognize the major limitations to stepwise selection approaches, but we did take steps to reduce the likelihood of identifying spurious relationships. Most notably, we avoided the common practice of throwing many predictors into an automated selection procedure to “see what sticks”. Our potential predictor variables were selected based on prior knowledge, and we carefully evaluated the influence of variable removal on the overall model at every step. Note that when variables were fitted together in a multivariable model, the adjustment for climate effects rendered all other variables insignificant, and the removal of each variable had no meaningful influence on the coefficients or p-values of other variables. For added transparency, we added the results of the maximal global model to the results section, which shows that only the climate variables had a significant association with the outcome after controlling for all additional covariates.

Regarding your suggestion of a multi model approach, we re-analyzed the data by fitting multiple models and ranking them based on AIC. We fitted models under different hypotheses, grouping variables by land cover (percent forest in 2019 and land cover change from 2001-2019), sociodemographic factors, and climate. We fitted models that only included each variable category, a model with every variable, and models with each pairwise combination of predictor categories. The same model that we identified with backwards elimination (climate only model) had the lowest AIC. We did not include these results in the revised manuscript.

**Minor Comments**

Minor point for organization that echoes earlier comment about Methods…in the Methods section I recommend putting the Software paragraph after the description of statistical methods. As is, implementation is given for methods that haven’t been introduced yet, and it breaks up the case definition and case data sections (which you may be able to just combine under one heading anyway).

We removed the software section and combined the case data and case definition sections.

This was probably an error generated in the reviewer pdf, but Figure 1 was not included in the document.

L84 Don’t think I’ve seen the term “accessory vectors” used. Consider using “secondary” instead?

We revised this sentence.

L95 Are these estimated total annual costs? Or lifetime management per case?

We specified that this is the potential cost for management (acute treatment and management of long-term sequelae) for a single case.

L103 Might be useful to mention that Ae. albopictus and Ae. japonicus are invasive here

Thank you. We revised this sentence to state that these are invasive vectors.

L135 Consider using term other than hotspot here

We revised this sentence to use different terminology.

L163 I think it would be helpful to show the difference in probable and confirmed case totals. If not in the main manuscript, a graph or table showing these totals by year/region would be worth adding to supplemental materials.

The datasets provided by the departments of health did not include probable vs. confirmed.

West Nile virus is mentioned as another endemic arboviral disease in the area. Are cases frequently reported in the study region?

West Nile virus infections are reported in the study area, but with lower frequency than LACV. WNV is the most common cause of mosquito-borne disease in Tennessee and North Carolina, but LACV infections are more common in the study area of eastern TN and western NC.

L181 Data were provided to home address. Need to describe aggregation of cases to zip codes.

We added a sentence describing the aggregation of data by ZIP codes.

L192 Were there any changes in zip code boundaries during the study period?

Yes. We accounted for this by using time standardized ZCTAs (i.e., populations were standardized to 2010 ZCTA boundaries). We added a small amount of additional text to clarify this.

L205 Jenks classification (not Jenk’s). Also provide a citation for use of Jenks classification for health data visualization (CDC has one, I believe)

We provided a citation for Jenks natural breaks.

L211 Specify if scan statistic was run on cases or rates (looks like rates, but need to specify)

The scan statistics use cases and the total population, effectively modeling risk. We elaborated on this in the revised text.

L212 Was the spatial scan statistic run as two separate analyses, or is this saying that two different population denominators were used for respective time periods in the same analysis? Unclear as written.

The purely spatial cluster analyses were two separate models. We revised the text to further clarify this.

L230 Why run separate analyses for high and low clusters? Looks like it’s because of different thresholds on cluster size for high versus low. Specify in text, and provide citations for these cutoffs.

Yes, separate analyses were run in order to use different cluster size thresholds. This was explained in the manuscript – “For the high-risk cluster analysis, the maximum cluster size was specified to include no more than 5% of the total population in the study area to identify the most concentrated clusters to direct potential public health response. For the low-risk cluster analysis, the maximum cluster size included 20% of the population to avoid the identification of many small, adjacent clusters in large regions with excessively low risk.”

We added additional references to the SaTScan user guide. In this case low-risk clusters were very large, and smaller maximum population sizes resulted in the identification of many adjacent clusters. The high-risk clusters were relatively small regardless of population threshold, but most importantly, we aimed to identify focal areas for public health response, as stated.

L245 check wording of this sentence

Thank you for noting the awkward wording. This sentence included an error, which has been revised.

L268 “Data were”, check throughout

Corrected.

L269 Give versions for computer programs

The software versions were specified in the software section of the previous manuscript. We removed that section, so the revised manuscript now notes the software versions on first mention.

L298 Need to provide citations for variable selection process and threshold values

We had provided a citation earlier in the paragraph in the sentence that described the overall approach. Based on your comment we inserted that citation to sentences throughout the paragraph.

L439 Are there any hypothesized mechanisms for why transmission is so focally persistent?

We added a paragraph that addresses vertical transmission and stabilized infections LACV within *Aedes triseriatus* populations as a hypothesis for focal persistence.

Fig 4, suggest not using blue to depict clusters with elevated risk, as this color is often used to depict cold spots in other common hotspot analyses (and used this way in space-time analysis).

We revised this figure to only include shades of red to represent high-risk clusters.
